# Analysis of the Effectiveness of Removing Surface Defects by Brushing

**DOI:** 10.3390/ma15217833

**Published:** 2022-11-06

**Authors:** Jakub Matuszak, Kazimierz Zaleski, Krzysztof Ciecieląg, Agnieszka Skoczylas

**Affiliations:** Department of Production Engineering, Mechanical Engineering Faculty, Lublin University of Technology, 20-618 Lublin, Poland

**Keywords:** defects, brushing, surface roughness, microhardness

## Abstract

The paper presents the results of a study on the effectiveness of removing surface defects by brushing. Damage to machine components usually begins on their surface or in the surface layer area. This determines the development of methods, conditions, and process parameters that will positively affect the stereometric and physical properties of the surface layer. Experiments were conducted in which surface defects were generated on a specially designed test stand. By controlling the load and speed of the defect generator it was possible to affect the geometry, depth, and width of the surface defect. A FEM simulation of the brushing treatment was carried out in order to determine the effect of fibers passing through a surface defect in the form of a groove with a small depth and width. It was shown that for certain conditions of brushing treatment, surface defects could be removed effectively. Moreover, the microhardness of the surface layer after the brushing process was analyzed. Changes in microhardness due to brushing reached up to 50 μm for EN AW-2024 aluminum alloy and up to 150 μm for AZ91HP magnesium alloy. The results demonstrated that brushing was an effective method for strengthening the surface layer and that the value of strengthening in the area of defects depended on the effectiveness of their removal.

## 1. Introduction

Damage to machine components predominantly begins on their surface or in the surface layer area. Consequently, new methods are developed to ensure process parameters and conditions that will positively affect the stereometric and physical properties of the surface layer. Surface defects can take different forms such as scratches, grooves, and burrs, and their form depends on the processing method used to manufacture a finished product. With elements subjected to fatigue loads, surface defects may act as a notch contributing to stress concentration and, consequently, to early failure of the element. Damage and failure mechanisms of crystalline materials were analyzed in [1]. In addition, surface defects that are left on the component in production, or formed during product operation have a negative effect on the aesthetic and decorative qualities of the finished products [2].

The term “surface layer” is used to describe the outer layer of an object that is located directly below its actual surface, including the surface as well as a part of the volume of the object where chemical or physical properties have changed usually due to mechanical processing.

Defects that are formed during the manufacturing and further operation of a product may be characterized by large variations in shape, depth, or distribution on the product surface. The most important surface defects include scratches, grooves, cracks, craters, pitting, and burrs [3,4]. 

Scratch—is a narrow, linear indentation of a small depth on the surface of the workpiece, the cause of which usually results from the impact exerted on the surface by a sharp object. During machining, scratches can be formed due to inappropriate grinding conditions. 

Grooves—unlike scratches, are shallow linear indentations, with a large width relative to their depth, and their formation is caused by the impact of an external body indented into a given surface. Grooves can be formed when turning plastic materials with worn tools. 

Cracks are deep slots of a small width that are formed as a result of residual or external stresses. They can be a result of inappropriate thermal, casting, or welding conditions. Defects of this type may appear on the surface of the elements during its operation (as a result of material fatigue).

A crater is a circle-shaped depression, formed under certain conditions, for example, as a result of a spark discharge during EDM or as a result of hitting a surface with another object. A characteristic of craters is the presence of a flash, which is an annular area of material around the crater located above the surface of the material.

Pitting is a process in which irregularly shaped pits are formed due to either improper hardening conditions (hardening pits) or pitting corrosion.

Burrs are sharp protrusions in an irregularly shaped deformed material that are formed on the surface of an object near its edge. Burrs can be formed during machining, and sheet metal stamping (flash).

**Figure 1:**
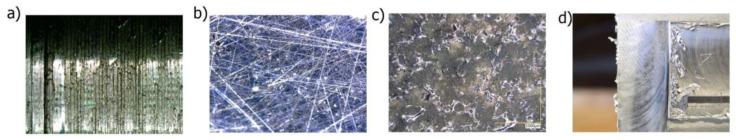
Examples of surface defects: (**a**) grooves, (**b**) scratches, (**c**) craters, (**d**) burrs.

Examples of selected surface defects are shown in Figure 1.

**Figure 2:**
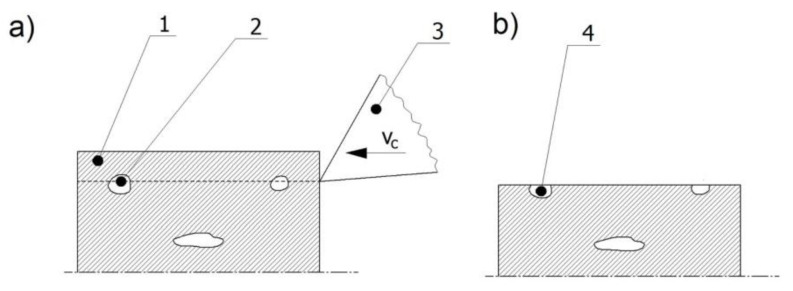
Formation of surface defects after machining a workpiece with internal defects (**a**) 1—machined layer, 2—internal defect, 3—tool, (**b**) 4—surface defect.

The formation of surface defects can be a result of internal defects or the removal of a layer of material (Figure 2). The workpiece has internal defects in the material before machining. When the cut layer is removed with a tool blade, the material defects are made visible on the machined surface as surface defects. Owing to the way they are manufactured, polymer composites are one example of materials that are prone to the formation of a higher number of internal defects. Improper bonding conditions of the reinforcing phase can affect the number of internal defects formed, and thus the strength of the composite material, its resistance to operating conditions, or damage to the final product [5]. There exist numerous methods for detecting both surface and internal defects. Among them are nonlinear techniques that use the cutting force signal for defect detection in milling, turning, or drilling processes. When the cutting edge passes through a surface defect, this causes a sudden change in the cutting conditions, which is reflected in the cutting force signal. Recurrence plots and recurrence quantification analysis are used to detect the areas where the cutting force signal is disturbed by the presence of surface defects [6,7,8]. 

Surface layer properties can be divided into two groups: stereometric and physical. Surface defects belong to the group of stereometric properties of the surface layer. Among the many ways and methods for changing the properties of the surface layer, one can distinguish burnishing and shot peening, based on cold work hardening of the surface layer [9]. The effect of burnishing treatment on the cold work hardening of the surface layer and thus improving its fatigue strength was investigated in [10,11,12,13].

Hybrid techniques such as laser-assisted methods are more and more widely used in industrial practice [14,15,16]. Laser energy can also be used to remove surface defects. The use of laser energy in combination with the classical cutting process causes the laser beam to be directed continuously at the workpiece surface to soften the cut layer and change the conditions of chip formation conditions. In turn, sequential support (with a time interval) causes a change in the microstructure of the surface layer of the workpiece [17]. 

There are many methods of removing surface defects. Most of them rely on mechanical effects on the surfaces of an object. One of the effective methods for removing defects is the brushing process. Typical processes based on brushing include deburring with simultaneous rounding of edges, glossing, as well as cleaning the surface, and removing old coatings, and corrosion; brushing is also used before the process of applying new coatings or adhesives. Studies of the deburring process by brushing have been presented in a number of papers that demonstrate the high efficiency of this process [18,19,20,21]. 

In [22], it was shown that after the water jet cutting process there are sharp edges and burrs mainly on the exit side of the jet from the cut material. Ceramic brushes were used to remove burrs and round-off edges. Depending on the stiffness of the tool and the processing parameters, it is possible to achieve rounding (for more flexible fibers) or chamfering (for stiff fibers) of the edges. Across the range of used parameters, brushing treatment has proven to be an effective method of deburring. In addition, the brushing process can be used to generate the required directionality of the geometric structure of the surface giving a decorative effect, or it can be used to generate the required properties of the surface layer [23]. 

The authors of [24,25] showed a 20% increase in fatigue life after brushing AA 5083-H111 aluminum alloy. The crack propagation of the fatigue-tested specimens was reduced mainly by increasing microhardness and inducing favorable compressive stresses due to the brushing treatment. 

Low forces that occur during brushing processing allow the application to thin-walled parts where low stiffness causes problems in the machining [26,27]. The use of appropriate machining strategies reduces the risk of deformation of thin-wall workpieces. However, after machining, the edges of thin walls require further processing involving deburring. 

A study [28] analyzed the effect of brushing on the corrosion resistance of AZ31B magnesium alloy. In the study, brushed magnesium alloy sheets with a thickness of 1 mm were used. A cup-type brush with a brush diameter and a wire thickness of 75 mm and 0.3 mm, respectively, was used in the study. A four-times increase in the corrosion resistance of the magnesium alloy after brushing treatment was observed. Increased corrosion resistance was also observed in the brushing of rebars [29]. Continuous surface nanocrystallization was achieved by wire brushing. The nanocrystallized layer was about 25 µm in thickness and had a grain size of 25 nm. In addition to that, the brushes are easy to use in automated machining because the flexible ends of the filaments that do the cutting work easily conform to the machined surface, sometimes shaped, without the need for costly tool positioning systems. 

The area of contact between the brush and the workpiece was analyzed in paper [30]. The authors used different types of brushes and a high-speed camera to visualize the deflection of the brush fibers throughout the contact zone with the workpiece. The popularity of using brushes as machining tools is due to their good performance, easy use in manual or automatic surface finishing, and uniform distribution of cutting forces on the surface of the workpiece. This allows for controlled material removal and simple workpiece clamping, as well as reduces the risk of damage to both the machine and the workpiece. Brushing treatment, due to the fact that it combines the features of machining, shot peening, and grinding for the removal of a layer of material, work hardening, or improvement of surface roughness, can therefore be referred to as a hybrid process [31,32,33,34,35,36]. 

Due to the insignificant amount of research in this area, the aim of this study is to investigate the effectiveness of removing surface defects by brushing, as well as to determine the microhardness distribution in the area of surface defects. 

## 2. Materials and Methods

Experiments were conducted on specimens of EN-AW-2024 aluminum alloy, and AZ91HP magnesium alloy. The test samples had the form of cuboidal plates with initial dimensions of 8 × 15 × 80 mm. The prepared samples were subjected to a face milling operation, using three-edge solid carbide cutter with a diameter of D = 20 mm, which was carried out with constant machining parameters (v_c_ = 500 m/min; f_z_ = 0.1 mm/tooth; a_p_ = 0.5 mm).

Table 1 shows the properties and chemical composition of the aluminum alloy. Increased strength values allow the use of this material in many industries, mainly in the aviation and automotive industries, where a high strength-to-density ratio is important.

Table 2 presents the properties and chemical composition of AZ91HP magnesium alloy. This cast magnesium alloy is widely used due to its relatively high strength combined with excellent corrosion resistance and good castability.

**Figure 3:**
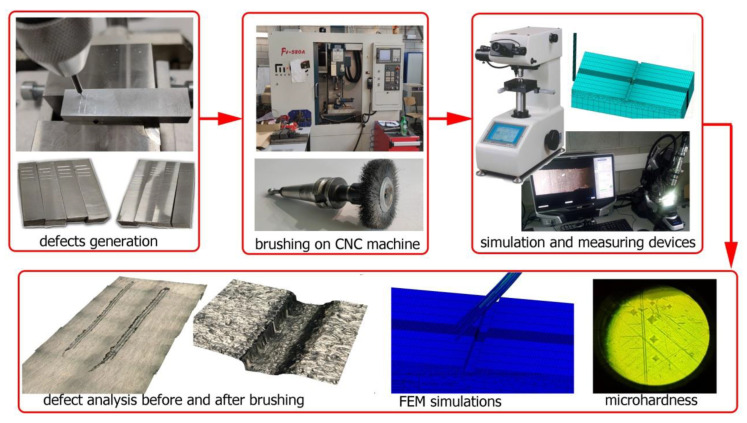
General diagram of the experiment methodology.

The overall experimental methodology is schematically shown in Figure 3. The brushing process was carried out on an FV580A vertical machining center.

**Figure 4:**
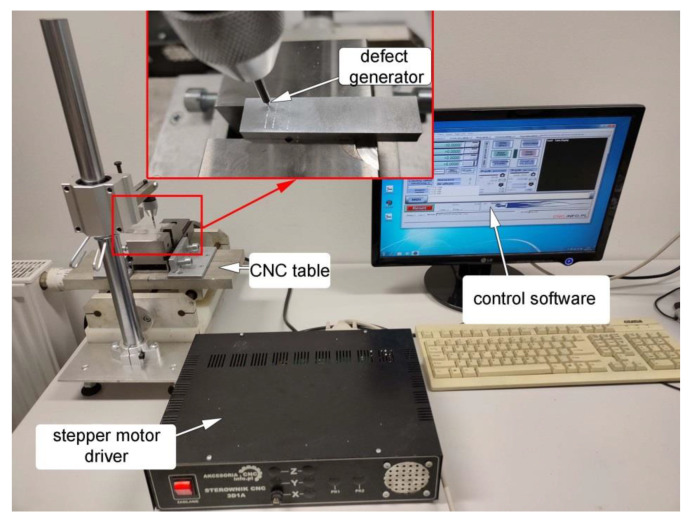
Test stand for generating surface defects.

Surface defects in the form of grooves were generated in the samples using a specially designed test stand that is shown in Figure 4. The test stand had a table with adjustable speed that could be controlled via stepper motor driver software. A carbide blade with an angle of 20 degrees was used as a defect generator. The speed of the defect generator was maintained constant at 500 mm/min. To vary the depth of surface defects, the defect generator blade was loaded with two force values: 5 N and 15 N. 

The brushing process was carried out using a 120 mm diameter roller brush with 0.2 mm thick steel fibers.

Table 3 shows the process conditions used in the experiment. The constant parameters of the brushing process were cutting speed and infeed, while the variables affecting process intensity were feed rate and the number of brushing passes. The research was carried out without the use of machining fluids.

**Figure 5:**
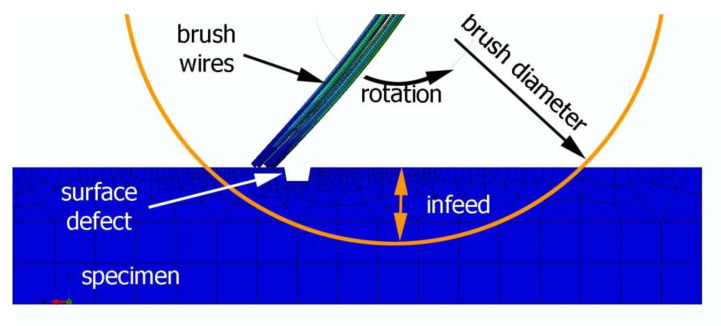
Simulation of the brushing process in Abaqus Explicit.

To visualize the passage of the brush fibers through the surface defect, a simulation reproducing the real process conditions was performed in Abaqus Explicit. For the numerical model of a 30 × 15 × 5 mm specimen, elements of type C3D8R were used. The element size in the zone where the brush fibers pass through the surface defect was reduced to 0.1 mm. For simulation, the Johnson–Cook constitutive model was used. The total number of elements in the mesh was 10,696, with a total of 13,427 nodes. The surface was hit by 9 steel fibers with a diameter of 0.2 and a length of 30 mm (which corresponded to the brush size). The total number of elements used for simulating the wire was 10,800 which amounted to 24,381 nodes. Figure 5 shows the simulation of positioning the modeled elements (specimen and wires). 

Both a visual analysis and measurements of the width and depth of surface defects were carried out with a Keyence VHX500 digital microscope (Keyence Ltd. HQ & Laboratories, Osaka, Japan). A x300 digital zoom and the technique of combining images were used.

**Figure 6:**
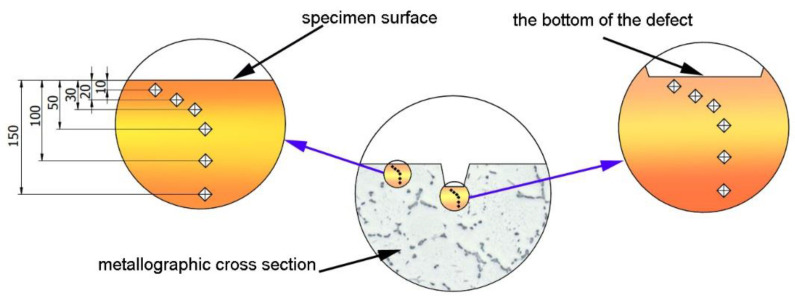
Methodology of microhardness measurement.

Microhardness was measured with the Leco LM700at device (LECO Corporation, St. Joseph, MI, USA) in compliance with the EN-ISO 6507-1:2018 standard. The measurements were made by the Vickers method, using a diamond indenter load of 10 g. A schematic illustrating the microhardness measurement methodology is shown in Figure 6. 

Microhardness was measured on metallographic specimens at the distances of 10, 20, 30, 50, 100, and 150 µm from the surface of the sample, as well as (using the same distances) from the surface of the bottom of the defect. The minimum distance between imprints was equal to 4 imprint sizes.

## 3. Results

### 3.1. Visual Defects Analysis before and after Brushing

**Figure 7:**
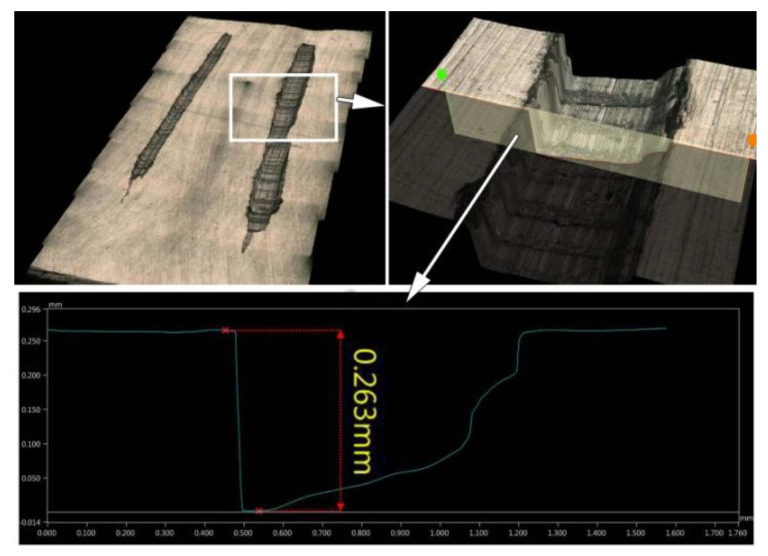
Measurement of surface defect depth.

Figure 7 shows the method of measuring the depth of surface defects formed in AZ91 HP alloy after the passage of the defect generator with a load of 15 N. The software of the microscope (the assembly function of a series of images) allows us to make a model and measure the depth in a section perpendicular to the surface of the test object. The average width of the grooves in AZ91 HP magnesium alloy inducted by the defect generator was 460 µm and 780 µm for a load of 5 N and 15 N, respectively. In turn, the average width of the grooves in EN AW-2024 aluminum alloy inducted by the defect generator was 200 µm and 375 µm for a load of 5 N and 15 N, respectively.

Table 4 shows a view of samples with surface defects (produced with a defect generator load of 5 N) after the brushing process conducted with the conditions established in the experiment. It can be observed that as the energy (intensity of the brushing process) increases, the efficiency of removing surface defects increases too.

For the EN AW 2024 alloy, the defects were removed during brushing conducted with a feed rate of v_f_ = 370 mm/min and five brushing passes. Table 5 shows the surface after brushing the defects produced with the 15 N load. In the images, one can observe a characteristic smoothing of one slope of the grooves after the passage of the brush fibers.

**Figure 8:**
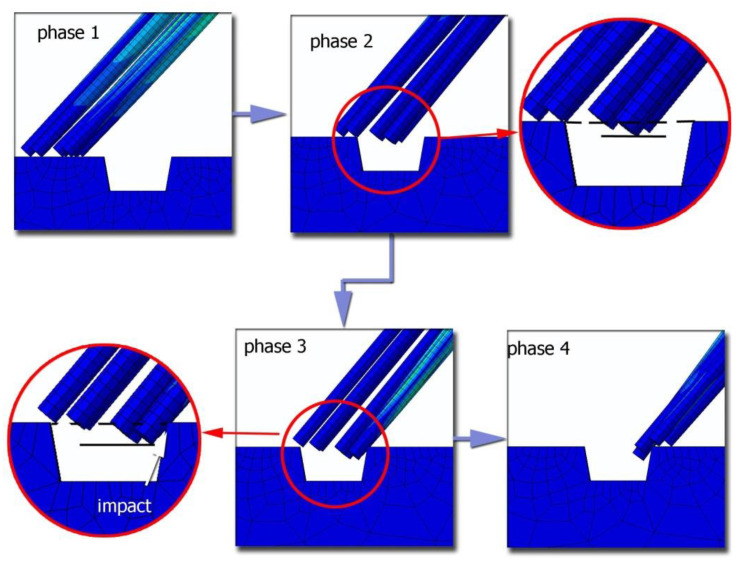
Simulation of the phases of fibers passing through a surface defect.

Figure 8 shows different phases of the passage of fibers through the surface defect obtained from the simulations conducted in Abaqus Explicit. In phase 1, the fibers glide across the surface of the workpiece, in phase 2 the first fibers fall into the groove (the fiber ends are below the line defining the workpiece surface), and in phase 3 the moment of first fibers hitting on the right slope of the surface defect occurs. As a result of the fibers passing below the line, defining the machined surface is an impact contact and smoothing of the slope of the surface defect, which can be seen in the real images taken with a microscope. On the other hand, in phase 4, the last fibers leave the defect area. 

**Figure 9:**
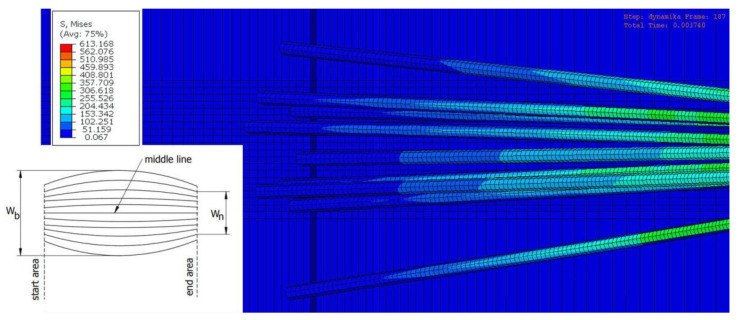
Top view simulation of the fibers passing through the surface defect.

Figure 9 shows the top view of the passage of fibers through a surface defect. Each fiber comes into contact with the workpiece in the start area and exits from the contact in the end area. During this time, the force of the fiber on the workpiece surface is not constant. The force depends on the angular position of individual fibers and the infeed value used in the experiment. The machining width W_b_ to which the sample is subjected is greater than the nominal brush width W_n_, which results from the forces acting perpendicular to the velocity vector. The width W_b_ primarily depends on the infeed value. 

### 3.2. Defects Depth before and after Brushing

**Figure 10:**
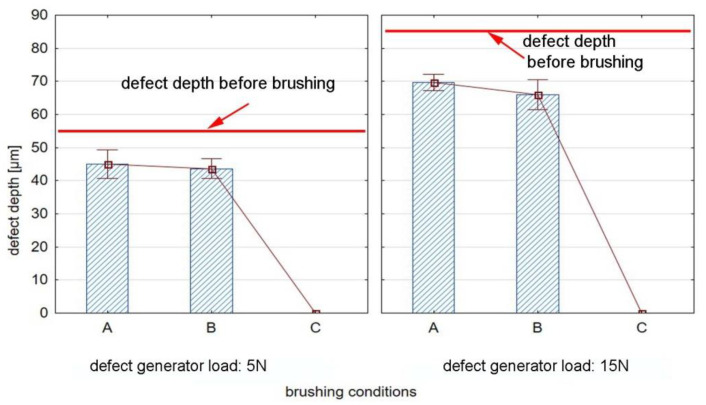
Effect of brushing conditions on defect depth for EN AW 2024 aluminum alloy.

Figure 10 shows the effect of brushing process conditions on the depth of the defects in EN-AW 2024 aluminum alloy specimens. The average depth of the grooves inducted by the defect generator was 55 µm and 85 µm for a load of 5 N and 15 N, respectively. 

For the conditions denoted by A and B (Table 3), the reduction in the defect depth after brushing is visible; however, defects after brushing are slightly smaller than before brushing. The depth of the groove for the defect generator force of 5 N decreased by 18% and 25% for conditions A and B, respectively, while the depth of the groove for the defect generator force of 5 N decreased by 18% and 22% for conditions A and B, respectively. On the other hand, the use of a feed rate of v_f_ = 370 mm/min and five brushing passes (conditions C), led to removing the defects from the surface of the workpiece. 

**Figure 11:**
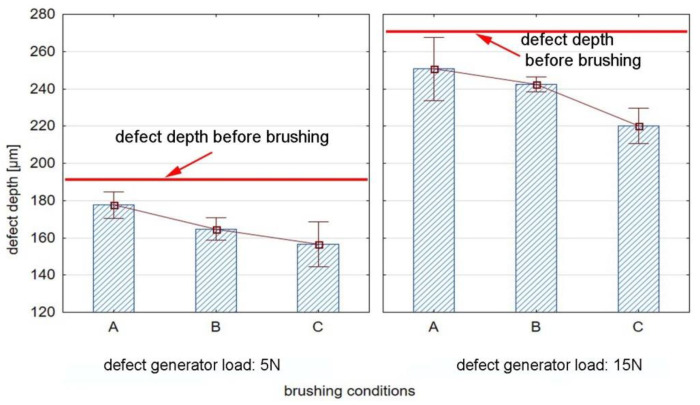
Effect of brushing conditions on defect depth for AZ91HP magnesium alloy.

Figure 11 shows the results of the brushing process conditions on the defect depth for the AZ91HP magnesium alloy. The average depth of the grooves after the passage of the defect generator was 193 µm and 270 µm for a load of 5 N and 15 N, respectively, which means that it was significantly higher than that obtained for the aluminum alloy.

For all tested process conditions, one can observe a reduction in the defect depth compared to the values before brushing. The depth of the groove for the AZ91HP decreased from 8% (condition A) to 18% (condition C) and in percentage was similar for both 5 and 15 N defect generator forces. As the energy is increased (by decreasing the feed rate or increasing the number of passes), the impact of the fibers on the workpiece surface is considerably greater and thus the defect depth is reduced.

### 3.3. Microhardness Analysis

**Figure 12:**
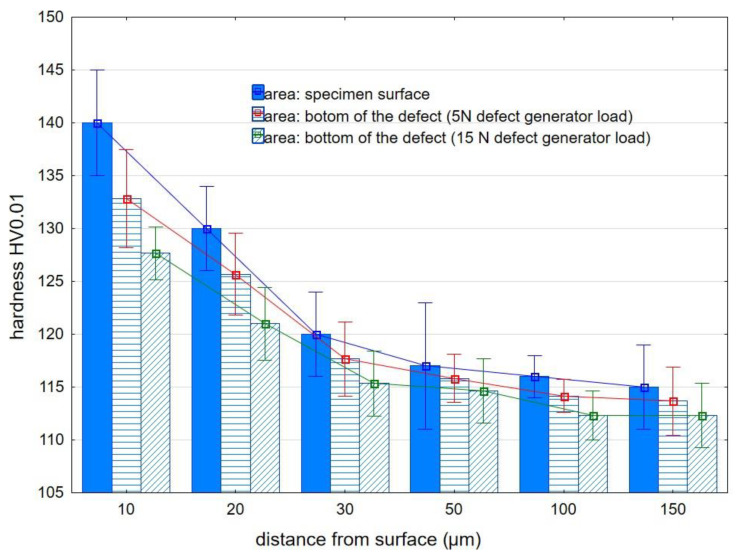
Microhardness distribution of EN AW 2024 aluminum alloy after brushing.

Microhardness distribution studies were carried out for constant brushing conditions (B conditions), i.e., v_c_ = 1265 m/min; v_f_ = 370 mm/min; infeed = 3 mm). Figure 12 shows the microhardness distribution in EN AW 2024 aluminum alloy.

**Figure 13:**
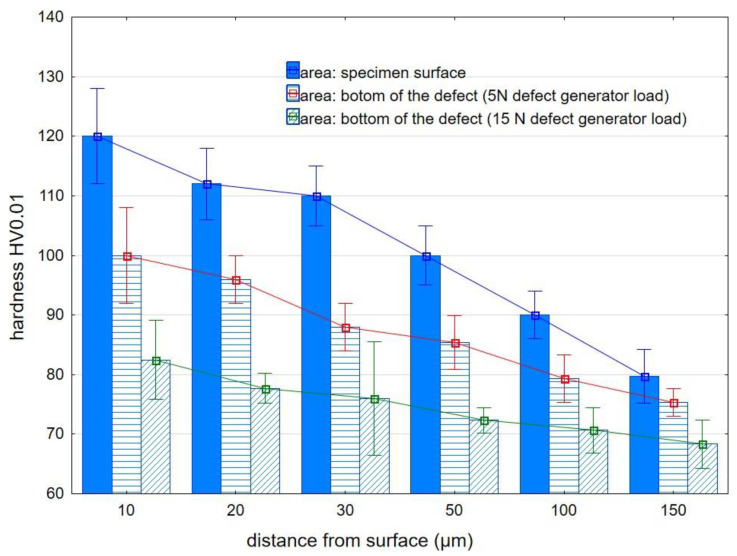
Microhardness distribution of AZ91 HP magnesium alloy after brushing.

The fibers that hit directly on the surface of the specimen cause the highest value of hardening of the surface layer (reaching HV0.01 = 140 at a distance of 10 µm from the surface). For the EN AW2024 alloy, the depth of the defects obtained in the experiment was significantly smaller compared to that obtained for AZ91HP magnesium alloy; therefore, the efficiency of their removal was higher (as shown in Table 4 and Table 5). As a result, there is a slight difference in the value of the microhardness of the surface layer below the bottom of the defect compared to the microhardness of the surface layer below the surface of the specimen. This is because the fibers exerted an impact on the bottom of the surface defect for a longer time (less energy was used to remove the defect yet more energy was required to harden the surface layer). Figure 13 shows the distribution of microhardness as a function of distance from the surface for AZ91 HP magnesium alloy. 

For a defect of smaller depth (produced with a 5 N defect generator load), some part of the fiber energy was used to remove the defect and the other was required for hardening the surface layer. In the case of a deeper defect (produced under a load of 15 N), the fibers in the first stage of machining did not hit the bottom of the groove to cause a slight strengthening of the surface layer in the area of the bottom of the defect in a later stage. Because the depth of the surface defects in the magnesium alloy was greater compared to that obtained for aluminum alloy, the effectiveness of their removal by brushing was lower, and the values of microhardness of the surface layer under the bottom of the defect were smaller, especially for the defects produced with a defect generator load of 15 N.

## 4. Conclusions

This study analyzed the effect of brushing process conditions on the effectiveness of removing surface defects. Defects in the form of grooves were generated in a controlled manner on a specially designed test station to ensure the repeatability of the process. The influence of the feed rate and the number of brushing tools passes on the possibility of removing surface defects from objects made of EN AW 2024 aluminum alloy and AZ91HP magnesium was investigated. The distribution of microhardness in the area of defects after brushing was also analyzed. The following conclusions summarize the results of the experimental study:

- The brushing treatment can be an effective method of reducing or removing surface defects;
- For aluminum alloy EN-AW 2024 using a feed rate of v_f_ = 370 mm/min and five brushing passes, surface defects were removed from the workpiece surface;
- AZ91HP magnesium alloy is characterized by a much greater depth of defects formed on the surface (both in the process of generating them and after the brushing process) compared to EN-AW 2024 aluminum alloy;
- Brushing treatment leads to the hardening of the surface layer.

## Figures and Tables

**Table 1:** Chemical composition and physical properties of EN-AW-2024 T351 aluminum alloy.

Chemical Composition, wt. %		Physical Properties	
Cu	3.8–4.9	Rm (MPa)	469
Mg	1.2–1.8		
Fe	≤0.5		
Si	≤0.5	Rp_0.2_ (MPa)	324
Mn	0.3–0.9		
Zn	≤0.25		
Zr + Ti	≤0.2	HB (Kgf/mm^2^)	110
Cr	≤0.1		
Other	0.2		
Al	Rest		

**Table 2:** Chemical composition and physical properties of AZ91HP magnesium alloy.

Chemical Composition, wt. %		Physical Properties	
Al	9.45	Rm (MPa)	200
Zn	0.72		
Ni	0.025		
Mn	0.017	Rp_0.2_ (MPa)	135
Cu	0.016		
Si	0.03		
Fe	0.002	HB (Kgf/mm^2^)	65
Mg	Rest		

**Table 3:** Technological parameters of brushing.

Velocity [m/min]	Feed Rate [mm/min]	Infeed [mm]	Number of Passes	Experimental Test
1265	1000	3	1	A
	370		1	B
	370		5	C

**Table 4:** View of defects after the brushing process (for a defect generator load of 5 N).

	Brushing Conditions		
	A	B	C
AZ91 HP	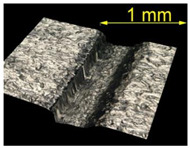	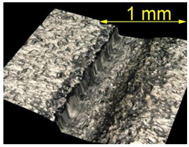	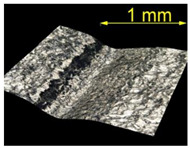
EN AW 2024	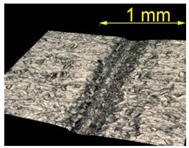	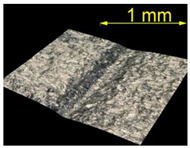	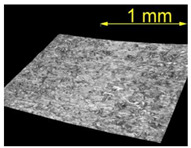

**Table 5:** View of defects after the brushing process (for a defect generator load of 15 N).

	Brushing Conditions		
	A	B	C
AZ91 HP	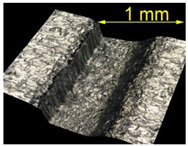	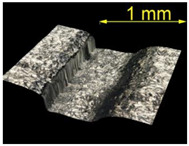	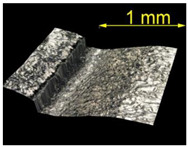
EN AW 2024	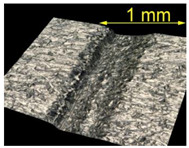	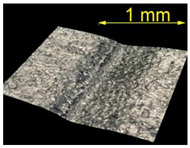	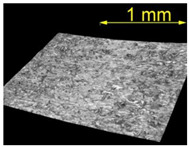

## Data Availability

The data presented in this study are available on request from the corresponding author.
